# 98. COVID-19 Vaccine Breakthrough Hospitalizations and Deaths in the Veterans Health Administration

**DOI:** 10.1093/ofid/ofab466.098

**Published:** 2021-12-04

**Authors:** Gina Oda, Aditya Sharma, Cynthia A Lucero-Obusan, Patricia Schirmer, Mark Holodniy, Connor W Edson, Ramin Khaksar

**Affiliations:** 1 Department of Veterans Affairs, Palo Alto, CA; 2 Stanford, San Carlos, California

## Abstract

**Background:**

A COVID-19 vaccine breakthrough infection is defined as SARS-CoV-2 RNA or antigen detected ≥ 14 days after completion of a final vaccine dose. CDC’s May 25 MMWR report of 10,262 vaccine breakthrough infections in the U.S. is likely an underestimate. Herein, we report Veterans Health Administration (VHA) breakthrough cases, focusing on hospitalizations and deaths.

**Methods:**

We extracted COVID-19 vaccine breakthrough infections tested between 1/19/2021 and 4/30/2021 from the VHA Corporate Data Warehouse (including screening tests). We reviewed medical records of cases who died and/or were hospitalized within 14 days of SARS-CoV-2 positive test for clinician documentation of conditions deemed high risk for COVID-19 and to confirm hospitalization or death was related to COVID-19. SARS-CoV-2 whole genome sequencing (Clear Labs platform) and antigen testing (Abbott BinaxNOW) from available patient samples were performed and Pangolin lineage determined.

**Results:**

1,142 COVID-19 vaccine breakthrough infections were identified. 357/1,142 (31.3%) were hospitalized and/or died. 1,085 (95%) were male (Table 1), and median age was 72.5 years (74 years for hospitalized/deceased patients). COVID-19 infection contributed to hospitalization and/or death in 139 (38.9%) cases. The remaining 218 (61.1%) were hospitalized or died of causes apparently unrelated to COVID-19. Smoking and heart conditions were seen most frequently among hospitalized/deceased breakthrough cases (Table 2). Variant B.1.1.7 was predominant, present in 17/27 (63%) total samples sequenced, and 13/21 (61.9%) hospitalized/deceased. (Table 3). Of 21 sequenced hospitalized/deceased cases, SARS-CoV-2 antigen positivity was present in 11 (52.4%).

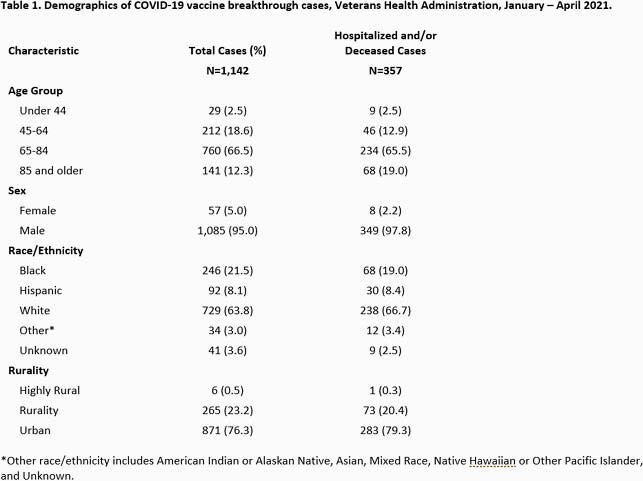

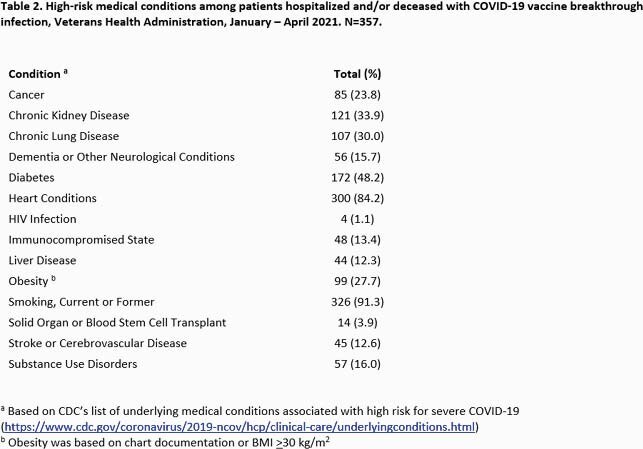

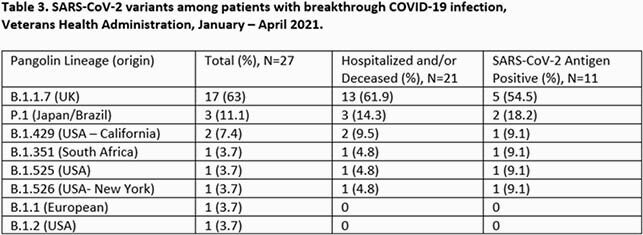

**Conclusion:**

Compared to CDC reported breakthrough infections, VHA cases were more male, older, and hospitalized/died at higher frequency. Further study is needed to determine the contribution of specific underlying conditions, COVID-19 vaccine formulations and variants on hospitalization and death among COVID-19 vaccine breakthrough infections. Sequencing efforts for breakthrough cases should be intensified, particularly for those presenting with more severe infections.

**Disclosures:**

**All Authors**: No reported disclosures

